# Clinical relevance of ErbB-2/HER2 nuclear expression in breast cancer

**DOI:** 10.1186/1471-2407-12-74

**Published:** 2012-02-22

**Authors:** Roxana Schillaci, Pablo Guzmán, Florencia Cayrol, Wendy Beguelin, María C Díaz Flaqué, Cecilia J Proietti, Viviana Pineda, Jorge Palazzi, Isabel Frahm, Eduardo H Charreau, Esteban Maronna, Juan C Roa, Patricia V Elizalde

**Affiliations:** 1 Instituto de Biología y Medicina Experimental (IBYME), CONICET, Buenos Aires, Argentina; 2 Departamento de Anatomía Patológica (BIOREN) y, Universidad de La Frontera, Temuco, Chile; 3 Departamento de Cirugía, Facultad de Medicina, Universidad de La Frontera, Temuco, Chile; 4 Universidad Abierta Interamericana (UAI), Rosario, Argentina; 5 Servicio de Patología, Sanatorio Mater Dei, Buenos Aires, Argentina; 6 Laboratory of Molecular Mechanisms of Carcinogenesis, Instituto de Biología y Medicina Experimental (IBYME), Obligado 2490, Buenos Aires 1428, Argentina

## Abstract

**Background:**

The biological relevance of nuclear ErbB-2/HER2 (NuclErbB-2) presence in breast tumors remains unexplored. In this study we assessed the clinical significance of ErbB-2 nuclear localization in primary invasive breast cancer. The reporting recommendations for tumor marker prognostic studies (REMARK) guidelines were used as reference.

**Methods:**

Tissue microarrays from a cohort of 273 primary invasive breast carcinomas from women living in Chile, a Latin American country, were examined for membrane (MembErbB-2) and NuclErbB-2 expression by an immunofluorescence (IF) protocol we developed. ErbB-2 expression was also evaluated by immunohistochemistry (IHC) with a series of antibodies. Correlation between NuclErbB-2 and MembErbB-2, and between NuclErbB-2 and clinicopathological characteristics of tumors was studied. The prognostic value of NuclErbB-2 in overall survival (OS) was evaluated using Kaplan-Meier method, and Cox model was used to explore NuclErbB-2 as independent prognostic factor for OS.

**Results:**

The IF protocol we developed showed significantly higher sensitivity for detection of NuclErbB-2 than IHC procedures, while its specificity and sensitivity to detect MembErbB-2 were comparable to those of IHC procedures. We found 33.6% NuclErbB-2 positivity, 14.2% MembErbB-2 overexpression by IF, and 13.0% MembErbB-2 prevalence by IHC in our cohort. We identified NuclErbB-2 positivity as a significant independent predictor of worse OS in patients with MembErbB-2 overexpression. NuclErbB-2 was also a biomarker of lower OS in tumors that overexpress MembErbB-2 and lack steroid hormone receptors.

**Conclusions:**

We revealed a novel role for NuclErbB-2 as an independent prognostic factor of poor clinical outcome in MembErbB-2-positive breast tumors. Our work indicates that patients presenting NuclErbB-2 may need new therapeutic strategies involving specific blockage of ErbB-2 nuclear migration.

## Background

Human epidermal growth factor receptor 2 (ErbB-2/HER2), one of the members of the ErbB family of membrane receptor tyrosine kinases, is a major player in the breast cancer scenario [1]. Membrane ErbB-2 (MembErbB-2) overexpression is associated with poor clinical outcome [2]. At present, the recombinant humanized anti-ErbB-2 monoclonal antibody trastuzumab is successfully used for treatment of MembErbB-2-positive breast cancer in the metastatic and the adjuvant settings [3,4]. However, a significant percentage of tumors display primary or acquired trastuzumab resistance [5]. Notably, the dogma of ErbB-2 action as a membrane tyrosine kinase which induces the activation of mitogenic signaling pathways to promote breast cancer growth [1], has been challenged by the demonstration that MembErbB-2 migrates to the nuclear compartment, where it acts as a transcription factor (TF) [6]. Up to date, cyclooxygenase-2 (COX-2) gene is the only one whose expression has been shown to be modulated through the role of ErbB-2 as a TF in mammary tumor cells [6]. Correlation between ErbB-2 nuclear presence and COX-2 expression in breast tumor specimens has already been reported [6,7].

On the other hand, our recent findings have for the first time demonstrated that ErbB-2 acts also as a transcriptional coactivator [8]. We found that in the nucleus of breast cancer cells, ErbB-2 assembles a transcriptional complex in which it functions as a coactivator of the signal transducer and activator of transcription 3 (Stat3) to promote the expression of cyclin D1 [8], another gene known to induce breast cancer proliferation [9,10]. An exciting and novel finding of our study was the demonstration of the direct involvement of Nuclear ErbB-2 (NuclErbB-2) in breast cancer growth [8]. These findings led us to build our hypothesis that NuclErbB-2 presence could be associated with highly proliferative breast cancer subtypes which show a poor clinical outcome. Our present results have for the first time demonstrated that NuclErbB-2 is indeed a powerful and independent prognostic factor of poor clinical outcome in MembErbB-2-positive breast tumors.

## Methods

### Patients and Tissue Microarays (TMAs)

Paraffin-embedded tissue samples from 346 consecutively archived invasive breast carcinomas were selected for construction of TMA blocks from the files of the Histopathology Department of Temuco Hospital, Chile, from 1998 to 2006. From 273 patients, follow-up data was available for up to 13 years with a median follow-up time of 53 months. All patients were treated with surgery. This study was conducted with the approval of the Institutional Review Board on Human Research of Universidad de La Frontera (UF), and informed written consents were obtained from all patients before inclusion. The Board reviewed and approved the collection of tumor specimens, our survey data, and all clinical and pathological information as well as the restrospective biomarker analysis on anonymized specimens from the Temuco Hospital archival cohort. Pre-treatment patient staging was classified according to the American Joint Committee on Cancer (AJCC) system [11] through the Elston and Ellis histological grading system [12]. TMAs were constructed at the UF TMA Core Facility. In brief, H&E sections of all tumors were re-evaluated by a pathologist (PG) for suitability for TMA construction. Representative areas of tumor sections for each case were selected and circled to match the blocks for the tissue microarray. Blocks matching the circled slides were then retrieved to prepare the recipient block for the microarray. To assure the representation of selected cores, two areas of tumor sections per case were determined for assembly of the recipient blocks. Each target area on the selected blocks was punched to form a 2-mm-diameter tissue core, and placed consecutively on ~3 × 2 cm recipient blocks using a tissue microarrayer (Beecher Instrument, Silver Spring, MD). Tissue microarrays were then cut to 5 μm sections and placed on silane-coated glass slides, and the first and last slide were stained for H&E.

### Fluorescence in situ hybridization (FISH)

FISH was done according to PathVysion™ (Vysis, Inc, Downers Grove, IL) guidelines. ErbB-2 gene/chromosome 17 centromere signals ratio more than 2.2 was considered ErbB-2 amplification.

### Immunohistochemistry

Antigen retrieval was performed in 10 mM sodium citrate buffer pH 6 for 20 min at 96-98°C. Slides were incubated with primary antibodies as follows: c-erb-B2 clone A0485 (dilution 1:300 overnight at 4°C; Dako Carpinteria, CA), ErbB-2 clone C-18 (dilution 1:100, 1 h room temperature; Santa Cruz Biotechnology Inc., Santa Cruz, CA), RBT-HER2 (dilution 1:250, 1 h room temperature; Bio SB, Santa Barbara, CA), SP3 (dilution 1:100 overnight at 4°C; NeoMarkers, Fremont, CA). Sections were subsequently incubated with polydetector HRP system (Bio SB) and developed in 3-3'-diaminobenzidine tetrahydrochloride. Immunostainings were run with known positive and negative tumor controls and were blindly evaluated by two pathologists (PG and JCR) who ignored the results of FISH. ErbB-2 was scored according to the American Society of Clinical Oncology/College of American Pathologists (ASCO/CAP) guidelines [13]. Scores 2+ in which FISH confirmed ErbB-2 amplification, and 3+ were considered positive for MembErbB-2 overexpression. Estrogen (ER) and progesterone receptor (PR) were evaluated by IHC with clone 6F11 (Novocastra Laboratories, U.K) and clone hPRa2+hPRa3 (NeoMarkers, Freemont, CA), respectively and scored as described [14].

### Immunofluorescence

Antigen retrieval was performed by immersing the sections in 10 mM sodium citrate buffer pH 6 and microwaving at high power for 4 min. Slides were blocked in Modified Hank's Buffer (MHB) with 5% bovine serum albumin for 30 min and were incubated overnight at 4°C with the following ErbB-2 primary antibodies: C-18, raised against the ErbB-2 carboxy (C)-terminal region [8], 9G6, directed against the ErbB-2 amino (N) terminus, (1:50, Santa Cruz), e2-4001 raised against the ErbB-2 carboxy (C)-terminal region (1:50, Thermo Scientific, Pittsburgh, PA) and with phospho ErbB-2 (Tyr 877) and phospho ErbB-2 (Tyr 1221/1222) antibodies (1:50, Cell Signaling, Beverly, MA). Slides were then incubated with the corresponding Alexa 488-conjugated antibody (1:1000, Molecular Probes, Eugene, OR). Reduction of the autofluorescent background was performed by incubation with Sudan Black B 0.1% (Sigma-Aldrich, St. Louis, MO). Nuclei were stained with propidium iodide or DAPI (4',6-diamidino-2-phenylindole). Slides were analyzed by a Nikon Eclipse E800 confocal laser microscopy system. Negative controls were carried out with MHB instead of primary antibodies and using an ErbB-2 competitive peptide (Santa Cruz). NuclErbB-2-positive and -negative C4HD tumors from the model of mammary tumors induced by progestins were also used as controls [8]. Slides were independently scored by PG and JCR. Score discrepancies were re-evaluated and reconciled on a two-headed microscope. A third pathologist (EM) participated in IF staining and evaluation. MembErbB-2 and NuclErbB-2 expressions were evaluated in duplicate arrays.

### Statistics

Analyses were performed using STATA version 11 software (Stata Corp., College Station, TX). κ tests were applied to study concordance between MembErbB-2 levels determined with the different antibodies. Correlations between categorical variables were performed using the *χ*^2^-test or Fisher's exact test when the number of observations obtained for analysis was small. Specifically, Fisher's exact test was selected when the number of expected values was under five, because it uses the exact hypergeometric distribution to compute the *p*-value [15]. The *χ*^2^-test is basically an approximation of the results from the exact test, so few observations could potentially render erroneous results. Cumulative overall survival probabilities were calculated according to the Kaplan-Meier method, and statistical significance was analyzed by log-rank test. Multivariate analysis was performed using the Cox multiple hazards model. Adjustment for significant confounders was done to avoid increased bias and variability, unreliable confidence interval coverage, and problems with the model associated to the small size of our sample [16]. Variables included in the Cox model were those which resulted statistically significant (*p *< 0.05) in the log rank test (nuclear ErbB-2 staining, N, age and tumor grade). The remaining variables were excluded from our analysis (ER, PR, tumor size, clinical stage). All the tests of statistical significance were two-sided. *P *values < 0.05 were regarded as statistically significant. Guidelines for reporting tumor markers (REMARK) [17] were used, as outlined in Additional file 1.

## Results

### ErbB-2 nuclear localization in human breast cancers

**Figure 1:**
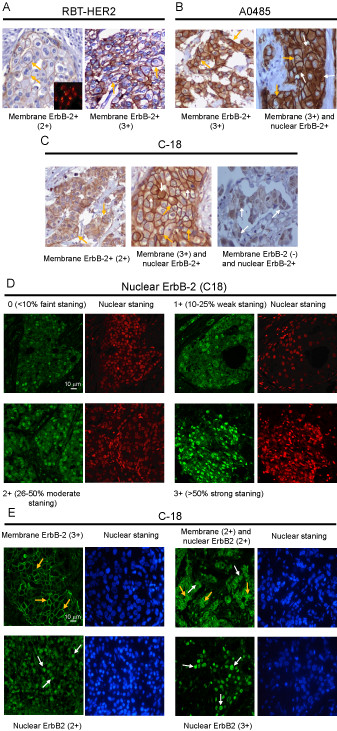
**Cellular localization of ErbB-2 in tumor samples**. **A**. IHC staining using RBT-HER2. Representative tumors with 2+ and 3+ scores of membrane ErbB-2 expression (400×). Inset shows FISH of ErbB-2 gene amplification in the tumor scored 2+ (1000×). **B**. IHC of ErbB-2 (400×) using A0485. Representative tumors showing only membrane ErbB-2 or membrane and nuclear ErbB-2 staining **C**. IHC of ErbB-2 (400×) using C-18. Examples of tumors showing exclusively membrane, membrane and nuclear or only nuclear positivity. **D and E**. IF staining of ErbB-2 with C-18. **D**. Nuclear ErbB-2 score. Nuclei were stained with propidium iodide (red). **E**. Examples of tumors showing only membrane, membrane and nuclear, and exclusive nuclear ErbB-2 expression (+2 and +3 scores). Nuclei were stained with DAPI (blue). In **A **to **C **and in **E **orange arrows indicate membrane ErbB-2 positivity and in **B**, **C**, and **E **white arrows indicate nuclear ErbB-2 presence. Membrane ErbB-2 levels in both IHC and IF were scored according to the American Society of Clinical Oncology/College of American Pathologists (ASCO/CAP) guidelines for ErbB-2/HER2 testing [13]. A score 0 represents no staining, 1+ weak, incomplete membrane staining in any proportion of tumor cells, 2+ complete membrane staining that is either non uniform or weak but with obvious circumferential distribution in at least 10% of tumor cells, or invasive tumors showing intense, complete membrane staining in 30% or fewer tumor cells, 3+ strong complete membrane staining in > 30% of tumor cells.

Since there are no previous reports on the clinical significance of NuclErbB-2 in breast cancer, we decided to tackle this issue first by conducting a retrospective study in a cohort of 273 primary invasive breast carcinomas from women living in Chile, a Latin American country. All these patients were treated with primary surgery. Our purpose was to assess the value of NuclErbB-2 as a prognostic marker within the context of the commonly used risk factors and the molecular subtype classification. The clinical and pathological characteristics of these specimens are shown in Table 1. TMAs from 226 tumor samples from our cohort were analyzed for MembErbB-2 expression by IHC using the rabbit monoclonal antibody RBT-HER2 and the rabbit polyclonal antibody A0485, which was approved by the Food and Drug Administration (FDA) and is routinely used for ErbB-2 evaluation [18]. We have recently detected MembErbB-2 and NuclErbB-2 in breast cancer cells by IF using the ErbB-2 rabbit polyclonal antibody C-18 [8]. Here we also assessed the ability of C-18 to detect ErbB-2 by IHC. MembErbB-2 levels were scored as previously described [13]. Scores of 3+ and only those of 2+ in which FISH confirmed ErbB-2 amplification were considered positive for MembErbB-2. Concordance between IHC scores of 2+ and FISH results were 92.1% (κ = 0.62) for RBT-HER2, 90.9% (κ = 0.61) for A0458, and 91.2% (κ = 0.61) for C-18. Our findings showed that 12.9% of the tumors showed MembErbB-2 overexpression with RBT-HER2, 13.8% with A0458, and 12.4% with C-18 (Table 2). Substantial concordance was found between 2+ and 3+ results obtained with RBT-HER2 and A0485 (91.9%, κ = 0.63), RBT-HER2 and C-18 (91.7%, κ = 0.64), and A0485 and C-18 (92.2%, κ = 0.64). Representative tumor samples stained with all three antibodies are shown in Figure 1A-C.

**Table 1:** Patient clinicopathological characteristics

Characteristic	N° patients	%
Total number of patients	273	
Age (years)		
Mean	55.5	
SD	13.7	
Menopausal status		
Premenopausal	111	41
Postmenopausal	162	56
Tumor size		
T1	58	21
T2	148	54
T3	45	17
T4	22	8
Lymph node status		
N0	118	43
N1	83	30
N2	72	27
Distant metastasis		
M0	256	94
M1	17	6
Metastatic sites location		
Bone	7	
Liver	3	
Skin	2	
Brain	2	
Not documented	3	
Clinical stage		
I	39	14
II	137	50
III	81	30
IV	16	6
Tumor grade		
1	45	17
2	134	50
3	86	33
Not documented	8	
Hormone receptor status, crossed results		
ER+PR+^a^	140	52
ER+PR-	50	18
ER-PR+	4	2
ER-PR-	75	28
Not documented	4	

^a^ER: estrogen receptor; PR: progesterone receptor

**Table 2:** Correlation between MembErbB-2 prevalence detected by immunohistochemistry

Antibody	MembErbB-2, n(%)		Total N (%)
		
	Negative	Positive	
**RBT-HER2**	175 (87.1)	26 (12.9)	201
**A0485**	181(86.2)	29 (13.8)	210
**C-18**	177 (87.6)	25 (12.4)	202

Because of the absence of some core sections on the slides, 201 patient samples could be analyzed for MembErbB-2 with RBT-HER2, 210 with A0485, and 202 with C-18. Scores of 3+ and only those of 2+ in which FISH confirmed ErbB-2 amplification were considered positive for MembErbB-2 overexpression

We next explored the nuclear presence of ErbB-2 by IHC. We did not find NuclErbB-2 in our tumors using RBT-HER2 (Figure 1A). Contrastingly, IHC with A0485 showed nuclear staining in some samples (Figure 1B), in accordance with seminal findings [6]. We also observed NuclErbB-2 in the tumors using C-18 (Figure 1C). To determine whether the difference in sensitivity to detect NuclErbB-2 between RBT-HER2 and the other two antibodies, A0485 and C-18, was due to an exclusive feature of RBT-HER2, we also stained the TMAs using SP3, another rabbit monoclonal antibody [18]. We found no nuclear staining in the tumor samples using SP3 (data not shown), suggesting that rabbit monoclonal antibodies may be less sensitive to detect NuclErbB-2 by IHC than rabbit polyclonal antibodies. Since we have most recently found that C-18 is exquisitely sensitive for the detection of MembErbB-2 and NuclErbB-2 in breast cancer cells by IF [8,19], we decided to assess ErbB-2 levels and cellular localization by IF in our TMAs. For this purpose, we first evaluated a series of commercially available total and phospho-ErbB-2 antibodies for their capacity to detect protein expression and activation by IF in paraffin-embedded tumor sections. As summarized in Table 3, we found that only C-18 was effective in detecting ErbB-2 protein expression. Levels of MembErbB-2 detected by IF were semiquantified using the same scores as those used in the IHC staining. On the other hand, an immunoreactivity score for NuclErbB-2 has not yet been established. Thus, we here scored NuclErbB-2 levels detected by IF considering both the percentage of ErbB-2 positive cells and staining intensity. A score of 0 represents faint or no staining in less than 10% of cells, 1+ weak nuclear staining in 10-25%, 2+ moderate staining in 26-50%, and 3+ strong staining in > 50% of cells (Figure 1D). Scores of 2+ and 3+ were considered positive for NuclErbB-2 presence. To assure the specificity of our results, C4HD tumors from the model of mammary carcinogenesis induced by progestins [20] were included in the TMAs. We recently showed a strong NuclErbB-2 localization in these tumors [8] that was completely abrogated by the transfection of a mutant ErbB-2 (hErbB-2ΔNLS) [21] unable to translocate to the nuclear compartment and which acts as a dominant negative inhibitor of endogenous ErbB-2 nuclear migration [8]. C4HD tumors were used as positive controls and C4HD tumors transfected with the hErbB-2ΔNLS as negative controls. Representative tumor samples are shown in Figure 1E. NuclErbB-2 positivity detected by IF in our cohort was 33.6% and no correlation was found between MembErbB-2 and NuclErbB-2 presence (Table 4). This is in contrast to previous findings using A0485 antibody in IHC or IF, which showed a direct correlation between MembErbB-2 and NuclErbB-2 positivity in breast tumors [6,7]. In order to assess whether this discrepancy might be due to differences in how NuclErbB-2 positivity was defined in those studies and in ours, we re-evaluated the correlation between MembErbB-2 and NuclErbB-2 using less stringent criteria for NuclErbB-2 positivity. We included tumors with 1+ score within the NuclErbB-2-positive group, but again found no significant correlation between MembErbB-2 overexpression and its nuclear presence (Table 4). We then added to our original cohort 73 patients. No correlation between MembErbB-2 and NuclErbB-2 presence was found in this larger cohort (Table 4). Having established a score for NuclErbB-2 positivity, we went back to our IHC staining of the arrays to semiquantify NuclErbB-2 levels. We found 4% NuclErbB-2 presence by IHC using A0485 and a significant correlation between MembErbB-2 and NuclErbB-2-positivity (*P *< 0.0001). On the other hand, the rate of NuclErbB-2 presence detected with C-18 was 12%, and we found no correlation between MembErbB-2 and NuclErbB-2 positivity (*P *= 0.07). Representative IHC staining pattern for ErbB-2 with C-18 is shown in Figure 1C. These findings demonstrate that indeed A0485 has lower sensitivity than C-18 for the detection of NuclErbB-2. Substantial to excellent overall concordance was found between MembErbB-2 positivity detected by IF and IHC with the different antibodies (Table 5). MembErbB-2 positivity showed an inverse relationship with the status of the steroid hormone receptors (HR) estrogen (ER) and progesterone (PR), as previously described [22] (Table 6).

**Table 3:** Comparison of ErbB-2 antibodies capacity to recognize ErbB-2 protein by immunofluorescence in paraffin-embedded tumor sections

	**Source**	**Isotype**	**Clone**	**Terminus**^**a**^	**MembErbB-2**	**NuclErbB-2**
	
***ErbB-2 antibodies***						
	Santa cruz Biotechnology	rabbit polyclonal	C-18	carboxy	Yes	Yes
		mouse monoclonal	9G 6	amino	No	No
	Thermo Scientific	mouse Monoclonal	e2-4001	carboxy	No	No
***Phospho ErbB-2 antibodies***						
phosphotyrosine 877	Cell Sinaling	rabbit polyclonal		carboxy	No	No
phosphotyrosine 1221/1222	Cell Signaling	rabbit polyclonal	6B12	carboxy	No	No

^a ^Localization of the epitope against which each ErbB-2 antibody was raised

**Table 4:** Correlation between NuclErbB-2 and MembErbB-2 expression studied by immunofluorescence with C-18 antibody

	MembErbB-2, n (%)		Total N (%)	*P value*
			
	Negative	Positive		
**NuclErbB-2 expression in the 226 patient cohort**				
Negative (0/1+)	126 (55.7)^a^	24 (10.6)	150 (66.4)	0.265^b^
Positive (2+/3+)	68 (30.0)	8 (3.7)	76 (33.6)	
Total N (%)	194 (85.8)	32 (14.2)	226 (100)	
Negative (0)	87 (38.5)	19 (8.4)	106 (46.9)	0.127
Positive (1+/2+/3+)	107 (47.4)	13 (5.7)	120 (53.1)	
Total N (%)	194 (85.8)	32 (14.2)	226 (100)	
**NuclErbB-2 expression in the 298 patient cohort**^**c**^				
Negative (0/1+)	171 (57.5)	30 (10.0)	201 (67.4)	0.176
Positive (2+/3+)	88 (29.5)	9 (3.0)	97 (32.6)	
Total N (%)	259 (86.9)	39 (13.1)	298 (100)	

^a ^Percentage of the total cohort. ^b ^Correlation between NuclErbB-2 and MembErbB-2 expression was determined by *χ*2 test. ^c ^We added to our original cohort 73 patients whose NuclErbB-2 and MembErbB-2 status by immunofluorescence staining we knew, but had not been included in our complete study because we lacked information regarding several clinicopathologic characteristics

**Table 5:** Concordance between detection of MembErbB-2 expression by immunofluorescence and immunohistochemistry

	MembErbB-2 (IF^a ^C-18), n (%)		Total N (%)	Overall concordance (%)	κ **statistics**^**d**^
				
	Negative	Positive			
**MembErbB-2 (IHC^b ^RBT HER2)**					
**Negative**	168 (83.4)^c^	7 (3.5)	175 (87.1)	96	0.84
**Positive**	1 (0.4)	25 (12.7)	26 (12.9)		
**MembErbB-2 (IHC A0485)**					
Negative	170 (80.9)	11 (5.2)	181 (86.2)	90	0.6
Positive	10 (4.8)	19 (9.1)	29 (13.8)		
**MembErbB-2 (IHC C-18)**					
Negative	168 (83.2)	9 (4.5)	177 (87.6)	93	0.7
Positive	5 (2.5)	20 (9.9)	25 (12.4)		

^a ^IF: Immunofluorescence. ^b ^IHC: Immunohistochemistry. ^c ^Percentage of the total cohort. ^d ^κ statistics (with a value of 1.0 indicating perfect agreement and a value of -1.0 indicating perfect disagreement) revealed excellent to substantial levels of concordance between detection of MembErbB-2 positivity by IF with C-18 and by IHC with RBT-HER2, A0485, and C-18 antibodies

**Table 6:** Correlation between estrogen and progesterone receptor status and MembErbB-2 expression

	MembErbB-2 (IF)^a^, n (%)		Total N (%)	*P *value	MembErbB-2 (IHC)b, n (%)		Total N (%)	*P *value
						
	Negative	Positive			Negative	Positive		
ER^c ^expression								
Negative	54 (23.90)^d^	20 (8.8)	74 (32.7)	0.001^e^	54 (26.8)	16 (8.0)	70 (34.8)	0.002
Positive	140 (61.9)	12 (5.3)	152 (67.3)		121 (60.2)	10 (5.0)	131 (65.2)	
PR^f ^expression								
Negative	90 (39.8)	24 (10.6)	114 (50.4)	0.003	88 (43.8)	20 (10.0)	108 (53.7)	0.011
Positive	104 (46.0)	8 (3.5)	112 (49.6)		87 (43.2)	6 (3.0)	93 (46.2)	

^a ^IF: Immunofluorescence staining performed with C-18 antibody. ^b ^IHC: Immunohistochemistry staining performed with RBT-HER2 antibody. ^c ^ER: Estrogen receptor. ^d ^Percentage of the total cohort. ^e ^Correlation between variables was determined by *χ*2 analysis. ^f ^PR: Progesterone receptor

### Association of NuclErbB-2 with risk factors and clinical outcome in breast cancer subtypes

**Figure 2:**
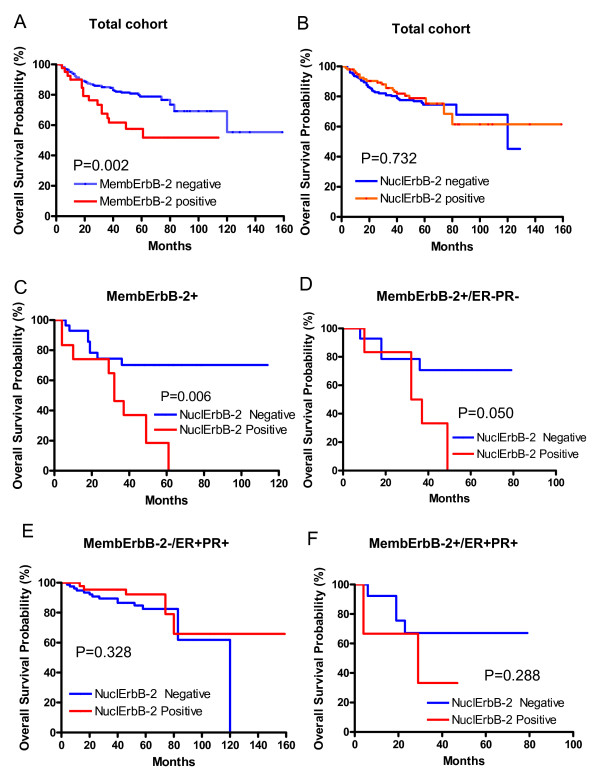
**Kaplan-Meier survival analysis and log-rank test were performed to correlate the levels of NuclErbB-2 with overall patient survival in the different breast cancer subtypes**. Kaplan-Meier analysis showing the correlation of MembErbB-2 overexpression with overall survival in our total cohort is shown in A.

We evaluated the relationship between NuclErbB-2 (2+ and 3+ scores) and the clinicopathological characteristics in 273 patients of our cohort. NuclErbB-2 was significantly associated with tumor size, lymph node positivity, and clinical stage (Table 7). As widely acknowledged in breast tumors [2], patients from our cohort bearing tumors with MembErbB-2 overexpression showed a significant worse overall survival (OS) than those lacking it (Figure 2A). Kaplan-Meier survival analysis showed no significant differences in OS between patients whose tumors expressed NuclErbB-2 and those with tumors lacking NuclErbB-2 (Figure 2B). We then explored the prognostic value of NuclErbB-2 in the group of patients that displayed MembErbB-2 overexpression. We found a significant association between NuclErbB-2 and the presence of distant metastasis at diagnosis (Table 7). In addition, patients bearing tumors with both MembErbB-2 and NuclErbB-2 had worse OS compared with patients whose tumors showed only MembErbB-2 (Figure 2C). In the molecular classification of breast cancer, tumors which lack ER and PR and overexpress MembErbB-2 (MembErbB-2+/ER-PR-) (Table 7) have been included in the ErbB-2-positive molecular subtype, associated with poor outcome [23-25]. Here we observed a significantly lower OS in the subset of patients with MembErbB-2+/ER-PR-tumors expressing NuclErbB-2 as compared to those whose tumors lack NuclErbB-2 (Figure 2D). Breast tumors expressing ER and PR and lacking MembErbB-2 (MembErbB-2-/ER+PR+), included in the luminal A molecular subtype [23-25], represent the subgroup with better prognosis of this study cohort. We found that the presence of NuclErbB-2 within this subgroup is significantly associated with larger tumor size, lymph node positivity, and higher clinical stages and grades (Table 7). However, we did not find significant differences in OS between patients whose tumors exhibited NuclErbB-2 and those lacking it (Figure 2E). Our cohort has a very small number (21) of patients with tumors MembErbB-2+/ER+PR+, a molecular signature of the recently defined as luminal-ErbB-2-positive subgroup [26]. Although a trend was observed for NuclErbB-2 presence and reduced survival in this subgroup (Figure 2F), the difference was not statistically significant (*P *= 0.288) because owing to limited sample size, the test was not powerful enough.

**Table 7:** Association between NuclErbB-2 expression and clinicopathological characteristics in breast cancer

	Total cohort (N = 273)			MembErbB-2+ N = 40			MembErbB-2+/ER-PR- (N = 21)			MembErbB-2-/ER+PR+ (N = 145)		
	
	NuclErbB-2, n (%)		*P *value	NuclErbB-2, n (%)		*P *value	NuclErbB-2, n (%)		*P *value	NuclErbB-2, n (%)		*P *value
								
	Negative	Positive		Negative	Positive		Negative	Positive		Negative	Positive	
Tumor size												
≤ 20 mm	46 (26.3)	13 (13.3)	0.012^a^	4 (14.8)	2 (16.7)	0.885^b^	2 (14.3)	1 (14.3)	1^b^	25 (31.6)	9 (13.8)	0.012^a^
> 20 mm	129 (73.7)	85 (86.7)		23 (85.2)	10 (83.3)		12 (85.7)	6 (85.7)		54 (68.4)	56 (86.2)	
Total N (%)	175 (64.0)	98 (36.0)		27 (69.2)	12 (30.8)		14 (66.7)	7 (33.3)		79 (54.9)	65 (45.1)	
Nodal metastasis												
Negative	82 (46.9)	33 (33.7)	0.034^a^	10 (35.7)	4 (33.3)	0.885^b^	7 (50.0)	2 (28.6)	0.642^b^	45 (57.0)	24 (36.4)	0.013^a^
Positive	93 (53.1)	65 (66.3)		18 (64.3)	8 (66.7)		7 (50.0)	5 (71.4)		34 (43.0)	42 (63.6)	
Total N (%)	175 (64.0)	98 (36.0)		28 (70.0)	12 (30.0)		14 (66.7)	7 (33.3)		79 (54.5)	66 (45.5)	
Distant metastasis												
M0	167 (95.4)	89 (90.8)	0.130^a^	27 (96.4)	9 (75.0)	0.049^b^	14 (100)	7 (100)		75 (94.9)	62 (93.9)	1^b^
M1	8 (4.6)	9 (9.2)		1 (3.6)	3 (25.5)		-	-		4 (5.1)	4 (6.1)	
Total N (%)	175 (64.0)	98 (36.0)		28 (70.0)	12 (30.0)		14 (66.7)	7 (33.3)		79 (54.5)	66 (45.5)	
Clinical stage												
I	31 (17.7)	8 (8.2)	0.031^a^	4 (14.3)	2 (20.0)	0.847^b^	2 (14.3)	1 (14.3)	1^b^	18 (22.8)	4 (6.1)	0.005^b^
II+III+IV	144 (82.3)	90 (91.8)		24 (85.7)	8 (80.0)		12 (85.7)	6 (85.7)		61 (77.2)	62 (93.9)	
Total N (%)	175 (64.0)	98 (36.0)		28 (73.7)	10 (26.3)		14 (66.7)	7 (33.3)		79 (54.5)	66 (45.5)	
Tumor grade												
Well to moderately differentiated^c^	121 (70.3)	57 (61.3)	0.134^a^	16 (59.2)	5 (45.5)	0.438^a^	9 (64.3)	3 (42.9)	0.397^b^	65 (83.3)	42 (68.9)	0.044^a^
Poorly differentiated	51 (29.7)	36 (38.7)		11 (40.8)	6 (54.5)		5 (35.7)	4 (57.1)		13 (16.7)	19 (31.1)	
Total N (%)	172 (65.0)	93 (35.0)		27 (71.0)	11 (29.0)		14 (66.7)	7 (33.3)		78 (56.1)	61 (43.9)	
ER^d ^expression												
Negative	53 (30.6)	26 (26.8)	0.540^a^	14 (51.9)	8 (72.7)	0.296^b^	14 (100)	7 (100)		1 (1.3)	2 (3.0)	0.457^b^
Positive	120 (69.4)	71 (73.2)		13 (48.1)	3 (27.3)		-	-		78 (98.7)	64 (97.0)	
Total N	173 (64.0)	97 (36.0)		27 (71.0)	11 (29.0)		14 (66.7)	7 (33.3)		79 (54.5)	66 (45.5)	
PR^e ^expression												
Negative	82 (47.4)	43 (44.8)	0.681^a^	18 (67.7)	9 (81.8)	0.452^b^	14 (100)	7 (100)		15 (19.0)	14 (21.2)	0.739^a^
Positive	91 (52.6)	54 (55.2)		9 (33.3)	2 (18.2)		-	-		64 (81.0)	52 (78.8)	
Total N	173 (64.0)	97 (36.0)		27 (71.0)	11 (29.0)		14 (66.7)	7(33.3)		79 (54.5)	66 (45.5)	

^a ^*χ*2 Test. ^b ^Fisher's exact test ^c ^Well to moderately differentiated: tumor grade 1 + 2, poorly differentiated: tumor grade 3. ^d ^ER: estrogen receptor

^e ^PR: progesterone receptor

Finally, multivariate analysis was performed using the Cox multiple hazards model. Adjustment for significant confounders was done to avoid increased bias and variability, unreliable confidence interval coverage, and problems with the model associated to the small size of our sample [16]. Variables included in the Cox model were those which resulted statistically significant (*p *< 0.05) in the log rank test (nuclear ErbB-2 staining, N, age and tumor grade). The remaining variables were excluded from our analysis (ER, PR, tumor size, clinical stage). Our findings demonstrated that NuclErbB-2 positivity was a significant independent predictor of worse survival in patients with MembErbB-2 (HR: 3.153, 95% CI: 1.118-8.891, *P *= 0.030). On the contrary, NuclErbB-2 presence did not have significant independent prognostic value in the overall patient population (HR:0.598, 95% CI:0.310-1.155, *P *= 0.126).

## Discussion

We here identify a novel role of NuclErbB-2 as a biomarker of poor clinical outcome in MembErbB-2-positive breast tumors.

First, we developed an IF protocol using the rabbit polyclonal antibody C-18, raised against the ErbB-2 carboxy (C)-terminal region [8], for the detection of ErbB-2 in paraffin-embedded tumor sections. We chose C-18 because we had already revealed its exquisite sensitivity to detect NuclErbB-2 and MembErbB-2 in breast cancer cells by IF [8,19]. Since seminal findings [6] and our own previous work [8] demonstrated that full-length ErbB-2 is present in the nuclear compartment of breast cancer cells, we also tested the mouse monoclonal 9G6 antibody directed against the ErbB-2 amino (N) terminus, which we previously found recognized NuclErbB-2 and MembErbB-2 by IF in breast cancer cells [8]. In addition, we used another ErbB-2 monoclonal antibody directed against the ErbB-2 C-terminus (clone e2-4001) as well as two polyclonal antibodies that recognize ErbB-2 phosphorylated at tyrosine 1222 or 877, two sites we have already revealed are phosphorylated in NuclErbB-2 [8]. We found that none of these antibodies detected NuclErbB-2 or MembErbB-2 by IF staining of the tissue arrays. This failure could be explained by the fact that we could not appropriately retrieve the single site or epitope on the antigen recognized by the monoclonal antibodies, or the site specifically containing the phosphorylated residue in the case of the two anti-phospho ErbB-2 polyclonal antibodies, due to the harsh conditions used in the preparation of the tissue blocks. On the contrary, given that polyclonal antibodies, such as C-18, recognize a broad range of epitopes on the antigen, including denaturation-resistant epitopes, we successfully detected ErbB-2 by IF in the tissue arrays with C-18.

Our present findings using the IF protocol with the C-18 antibody showed 33.6% NuclErbB-2 positivity and 14.2% MembErbB-2 overexpression in our Chilean cohort. This percentage of NuclErbB-2 staining is comparable to the one (38%) reported in breast tumors for nuclear expression of the epidermal growth factor receptor (EGF-R), another member of the ErbBs family [27]. Excellent to substantial levels of concordance were found between MembErbB-2 overexpression determined by IF with the C-18 antibody and by IHC using C-18, BT-HER2 or A0485 antibodies, indicating that the IF protocol we developed is as sensitive and specific as routinely used IHC staining procedures, including those using the Food and Drug Administration (FDA)-approved A0485 antibody, the same clone used in the commercial HercepTest immunostaining kit, also approved by the FDA. We found no correlation between NuclErbB-2 and MembErbB-2 positivity, in contrast to two previous works which reported a direct correlation between MembErbB-2 and NuclErbB-2 presence in breast tumors [6,7]. In said works, ErbB-2 localization was studied by either IHC [6] or IF [7] using the A0485 antibody. This discrepancy is unlikely due to differences in how NuclErbB-2 positivity was defined in those studies and in ours since we found the same results after we re-evaluated the correlation applying a less stringent criterion for NuclErbB-2 positivity. No correlation was found either when we extended the cohort under study. Another difference between said study exploring NuclErbB-2 prevalence in breast cancer by IF with A0485 [7] and ours, is the lower incidence of NuclErbB-2 detected by A0485 (12.3%) as compared to the one we found with C-18. A probable explanation for these disparities is that C-18 has much greater sensitivity for detecting NuclErbB-2 than A0485. Therefore, a significant number of samples which scored negative for NuclErbB-2 IF staining with A0485 are in fact detected by C-18, accounting for both the higher NuclErbB-2 positivity and the lack of correlation between MembErbB-2 and NuclErbB-2 presence. Several of our data support this hypothesis. First, our results of NuclErbB-2 incidence studied by IHC using A0485 and C-18 demonstrated that A0485 showed a significantly lower percentage of NuclErbB-2 presence (4%) as compared to C-18 (12%). Second, in accordance with the previous work mentioned above [6], we found a good correlation between MembErbB-2 overexpression and NuclErbB-2-positivity in the IHC staining with A0485, but no correlation between NuclErbB-2 and MembErbB-2 positivity in the IHC staining with C-18. Our recent findings demonstrating that in the nucleus of breast cancer cells ErbB-2 is associated with Stat3 and PR [8], could help to explain the different sensitivity of both antibodies. A larger number of epitopes recognized by A0485 could be blocked by NuclErbB-2 association with other proteins e.g. Stat3 and PR, than those recognized by C-18. On the other hand, the prevalence of NuclErbB-2 was lower when we used C-18 in IHC than when we used it in IF. This is likely due to the fact that while in the IHC staining with C-18 we used the standard conditions for membrane ErbB-2 detection [13], we introduced modifications in our IF protocol to enhance the sensitivity of NuclErbB-2 detection, including optimization of the antigen retrieval protocol and improvement of the conditions of interaction between antibodies and antigens, as described in a pioneering work assessing the clinical value of EGF-R nuclear presence [27]. The fact that the discrepancy in C-18 sensitivity when used in IF or in IHC is observed for the detection of NuclErbB-2 but not for MembErbB-2 argues for the possibility that differences in the conformation of MembErbB-2 and NuclErbB-2 and/or the association of NuclErbB-2 with other proteins may restrict antibody access to NuclErbB-2.

Although there are no extensive studies assessing the frequency of MembErbB-2 positivity in LA women in their own native countries [28], a series of startling works have shown the role of MembErbB-2 overexpression as predictor of poor outcome in breast cancer cohorts from Brasil [29,30]. Here we found 13% of MembErbB-2 overexpression by IHC and 14.2% by IF in our LA cohort. MembErbB-2-positive rates among Caucasian and Asian women, in whom most studies have been done to date, currently tend to be below 20%, with reports by most investigators stating that the real positive rate ranges between 15%-20% [31]. Therefore, our present findings for the first time show that the incidence of MembErbB-2 positivity in LA women living in their own countries is not significantly different from that of women living in developed countries.

MembErbB-2 overexpression has long been found to be associated with poor clinical outcome [2,31,32]. We here demonstrate that patients whose tumors expressed MembErbB-2 and NuclErbB-2 have worse OS compared with those with tumors showing only MembErbB-2. Moreover, NuclErbB-2 positivity was a significant independent predictor of poor survival in patients with MembErbB-2 overexpression. NuclErbB-2 also resulted a marker of lower overall survival in the subgroup of patients with tumors MembErbB-2+/ER-PR-, included in the ErbB-2-positive molecular subtype [23-25]. This latter finding for the first time highlights NuclErbB-2 presence as a molecular signature that might help to define two biologically distinct subsets of tumors within the ErbB-2-positive molecular subtype. The role of NuclErbB-2 as a TF [6], as well as our recent discoveries showing that ErbB-2 acts also as a coactivator [8] may underlie the poor outcome of tumors with NuclErbB-2. The combined capacity of ErbB-2 to activate mitogenic signaling pathways when located in the membrane and to act straight in the nucleus as a transcriptional regulator might drive the assembly of a gene network different from the one assembled in tumors with exclusive MembErbB-2 presence. This network would be involved in growth, metastasis and/or response to anti-ErbB-2 therapies, such as trastuzumab. Blockage of MembErbB-2 capacity to activate cytoplasmic signaling cascades is one of the mechanisms of trastuzumab action [5,33,34]. Therefore, a likely explanation to trastuzumab resistance could be ErbB-2 nuclear presence and function as a transcriptional regulator. Strong support to this possibility is provided by our findings that abrogation of ErbB-2 nuclear localization inhibits *in vitro *and *in vivo *growth of breast tumors expressing both NuclErbB-2 and MembErbB-2 [8].

The significant number of early stage tumors with no lymph node metastasis we have in our total cohort may account for the lack of NuclErbB-2 prognosis value in our overall population. Indeed although MembErbB-2 positivity is a poor prognostic factor in axillary node positive and negative tumors, the clinical significance of MembErbB-2 in early stage cancer is not yet completely understood [32,35-38]. On the other hand, our recent findings on PR and ErbB-2 interaction [8] provide a most exciting explanation for the comparable OS in MembErbB-2-/ER+PR+tumors with and without NuclErbB-2. We revealed that in HR+breast tumors, PR co-opts ErbB-2 function not only as membrane tyrosine kinase but also as transcriptional regulator [8]. In such scenario, endocrine therapies targeting ER, which in turn modulate the effects of PR, an ER-target gene, would also abolish ErbB-2 action at both membrane and nucleus.

Several key features of our cohort, such as levels of MembErbB-2 overexpression, the inverse correlation between HR presence and MembErbB-2 overexpression, and the fact that most of the tumors were in early stages, are comparable to those of the North American population [2,22,39] supporting extrapolation of our conclusions onto North American women. We hope our present findings will encourage further studies of NuclErbB-2 role as biomarker in larger populations stratified according to their treatment with endocrine therapy or chemotherapy and, in the case of MembErbB-2 overexpressing tumors, with trastuzumab or more recently with lapatinib.

## Conclusions

Our novel findings highlight the importance of developing new therapies to block ErbB-2 nuclear presence, such as our recent use of a mutant ErbB-2 that abolishes endogenous ErbB-2 nuclear migration [8].

## Abbreviations

MembErbB-2: Membrane ErbB-2; NuclErbB-2: Nuclear ErbB-2; LA: Latin American; IF: Immunofluorescence; IHC: Immunohistochemistry; OS: Overall survival; COX-2: Cyclooxygenase-2; Stat3: Signal transducer and activator of transcription 3; TMAs: Tissue microarrays; AJCC: American joint committee on cancer; ASCO/CAP: American Society of Clinical Oncology/College of American Pathologists; ER: Estrogen receptor; PR: Progesterone receptor; HR: Hormone receptors.

## Competing interests

The authors declare that they have no competing interests.

## Authors' contributions

PVE conceived and designed the study, analyzed data, and prepared the manuscript. RS participated in the study design, data collection, assembly and analysis of the data, and preparation of the manuscript. PG and JCR participated in the study design, data collection, assembly and analysis of the data, and performed the pathology and histology evaluations. FC, WB, MCDF, CJP, EHC, and VP participated in data collection. JP performed the FISH studies. EM performed IF evaluations. IF performed histology evaluations. All authors read and approved the final manuscript.

## Pre-publication history

The pre-publication history for this paper can be accessed here:

http://www.biomedcentral.com/1471-2407/12/74/prepub

## Supplementary Material

Additional file 1 **Table S1 **Study compliance with REMARK criteria. Click here for file
