# Supplementary material for: Clinical relevance of ErbB-2/HER2 nuclear expression in breast cancer
Source: BMC Cancer. 2012 Feb 22;12:74. doi: 10.1186/1471-2407-12-74 (PMC3342900; doi:10.1186/1471-2407-12-74)
Supplement: Additional file 1 — Table S1 Study compliance with REMARK criteria. [file 1471-2407-12-74-S1.PDF]

Supplementary Table 1 Study compliance with REMARK criteria

INTRODUCTION	1. The marker examined, study objectives, and hypotheses are stated in the Background section.
MATERIALS AND METHODS	**Patients**<br>2. The characteristics of study patients are detailed in Table 1. Inclusion criteria are stated in the Methods section.<br>3. Treatment information available indicated that all patients were treated with surgery as detailed in the Methods section<br>**Specimen characteristics**<br>4. Formalin-fixed paraffin-embedded material was used, which was stored at the Histopathology Department of Temuco Hospital, Chile, from 1998 to 2006 for construction into tissue microarrays.<br>**Assay methods**<br>5. Details of protocols used for immunohistochemistry, immunofluorescence, and FISH are provided under "Methods". Scoring of immunohistochemical and immunofluorescence markers was conducted blinded to survival time data, patient, and tumor characteristics.<br>**Study design**<br>6. Patients were recruited retrospectively utilizing anonymised archival material as specified in the Methods section. The time period from which cases were recruited is also specified under "Methods".<br>7. The clinical endpoint used is defined in the Methods section.<br>8. All candidate variables considered and included in statistical models are specified in the Methods section.<br>9. Sample size provided sufficient statistical power to address pre-specified hypotheses.<br>**Statistical analysis methods**<br>10. The Methods section contains details on statistical methods used, model-building procedures and testing of assumptions. The multivariate analysis using the Cox multiple hazards model was conducted on a sample of 40 patients, therefore we carried out the adequate control of the confounding factors described under "Methods" in order to avoid increased bias and variability, unreliable confidence interval coverage, and problems with the model. Correlations between categorical variables were performed using the χ^2-test or Fisher's exact test when the number of observations obtained for analysis was small. Specifically, Fisher's exact test was selected when the number of expected values was under five, because it uses the exact hypergeometric distribution to compute the p-value. The χ^2-test is basically an approximation of the results from the exact test, so few observations could potentially render erroneous results.<br><br>*11. Details of how marker values were handled and cutpoints determined are shown in the Methods section.*
RESULTS	**Data**<br>12. The numbers of patients included at each stage and within subgroups are detailed in the results section**.** The number of events in the multivariate analysis is stated in the Results section.<br>13. Characteristics of study participants are detailed in Table 1.<br>**Analysis and presentation**<br>14. The relationship of NuclErbB-2 to standard prognostic variables is detailed in the Results section (Table 7).<br>15. Univariate analyses for NuclErbB-2 in the different subgroups of patients are shown in Figure 2 and described in the text.<br>16. Multivariate analysis for NuclErbB-2 including estimated effect size (hazard ratio) and confidence interval is described in the text. All variables included in the model were those that resulted statistically significant ($p<0.05$) in the log rank test, as described in the text.<br>17. Internal validation of the use of IF staining with the C-18 antibody for the detection of NuclErbB-2 and MembErbB-2 using an enhanced cohort is described in the text.
DISCUSSION	19. The study results were discussed in the context of our pre-specified hypothesis and other relevant studies.<br>20. The clinical value and implications for future research were stated.
